# Prognostic Value of the BIO-Ra Score in Metastatic Castration-Resistant Prostate Cancer Patients Treated with Radium-223 after the European Medicines Agency Restricted Use: Secondary Investigations of the Multicentric BIO-Ra Study

**DOI:** 10.3390/cancers14071744

**Published:** 2022-03-29

**Authors:** Matteo Bauckneht, Sara Elena Rebuzzi, Marta Ponzano, Roberto Borea, Alessio Signori, Viviana Frantellizzi, Elisa Lodi Rizzini, Manlio Mascia, Valentina Lavelli, Alberto Miceli, Maria Silvia De Feo, Antonio Rosario Pisani, Susanna Nuvoli, Vincenzo Tripoli, Alessio Giuseppe Morganti, Paolo Mammucci, Salvatore Caponnetto, Guglielmo Mantica, Angelo Domenico Di Nicola, Carlo Villano, Luca Cindolo, Silvia Morbelli, Gianmario Sambuceti, Stefano Fanti, Renato Patrizio Costa, Angela Spanu, Giuseppe Rubini, Fabio Monari, Giuseppe De Vincentis, Giuseppe Fornarini

**Affiliations:** 1 Department of Health Sciences (DISSAL), University of Genova, 16132 Genova, Italy; ponzano.marta@gmail.com (M.P.); alessio.signori.unige@gmail.com (A.S.); albertomiceli23@gmail.com (A.M.); silviadaniela.morbelli@hsanmartino.it (S.M.); sambuceti@unige.it (G.S.); 2 Nuclear Medicine, IRCCS Ospedale Policlinico San Martino, 16132 Genova, Italy; 3 Medical Oncology, Ospedale San Paolo, 17100 Savona, Italy; saraelena89@hotmail.it; 4 Department of Internal Medicine and Medical Specialties (Di.M.I.), University of Genova, 16132 Genova, Italy; 5 Medical Oncology Unit 1, IRCCS Ospedale Policlinico San Martino, 16132 Genova, Italy; roby.borea@gmail.com (R.B.); giuseppe.fornarini@hsanmartino.it (G.F.); 6 Department of Radiological Sciences, Oncology and Anatomical Pathology, Sapienza University of Rome, 00185 Rome, Italy; viviana.frantellizzi@uniroma1.it (V.F.); mariasilvia.defeo@uniroma1.it (M.S.D.F.); giuseppe.devincentis@uniroma1.it (G.D.V.); 7 Radiation Oncology, IRCCS Azienda Ospedaliero-Universitaria di Bologna, 40138 Bologna, Italy; elisalodirizzini@libero.it (E.L.R.); alessio.morganti2@unibo.it (A.G.M.); fabio.monari2@unibo.it (F.M.); 8 Unit of Nuclear Medicine, Spirito Santo Hospital, 65100 Pescara, Italy; manlio.mascia@ausl.pe.it (M.M.); angelodomenico.dinicola@asl.pe.it (A.D.D.N.); carlo.villano@asl.pe.it (C.V.); 9 Nuclear Medicine Section, Interdisciplinary Department of Medicine, University of Bari “Aldo Moro”, 70124 Bari, Italy; valentina.lavelli@gmail.com (V.L.); apisani71@libero.it (A.R.P.); paolo.mammucci@outlook.com (P.M.); giuseppe.rubini@uniba.it (G.R.); 10 Unit of Nuclear Medicine, Department of Medical, Surgical and Experimental Sciences, University of Sassari, 07100 Sassari, Italy; snuvoli@uniss.it (S.N.); aspanu@uniss.it (A.S.); 11 Unit of Nuclear Medicine, Biomedical Department of Internal and Specialist Medicine, University of Palermo, 90100 Palermo, Italy; vincenzo.tripoli@policlinico.pa.it (V.T.); renatopatrizio.costa@gmail.com (R.P.C.); 12 Department of Experimental, Diagnostic, and Specialty Medicine-DIMES, Alma Mater Studiorum Bologna University, 40138 Bologna, Italy; stefano.fanti@aosp.bo.it; 13 Medical Oncology Unit B, Policlinico Umberto I, Sapienza University of Rome, 00185 Rome, Italy; salvo.caponnetto@uniroma1.it; 14 Department of Urology, IRCCS Ospedale Policlinico San Martino, 16132 Genova, Italy; guglielmo.mantica@hsanmartino.it; 15 Department of Urology, Villa Stuart Private Hospital, 00185 Rome, Italy; lucacindolo@virgilio.it; 16 Nuclear Medicine Unit, IRCCS Azienda Ospedaliero-Universitaria di Bologna, 40138 Bologna, Italy

**Keywords:** metastatic castration-resistant prostate cancer, Radium-223, inflammatory indices, neutrophil-to-lymphocyte ratio, clinical factors, treatment completion, European Medicines Agency restricted use

## Abstract

**Simple Summary:**

Radium-223 prolongs overall survival in metastatic castration-resistant prostate cancer patients with bone metastases. However, prognosis after Radium-223 administration varies among patients. One possible reason for this heterogeneity could rely on the suboptimal selection of patients with unfavourable prognostic factors. Moreover, in 2018, the European Medicines Agency limited the Radium-223 prescription to patients pre-treated with at least two systemic therapies or ineligible for any systemic treatment and more than six bone lesions. This moved Radium-223 treatment to the later stages of the disease, making the patient selection process even more challenging. In the BIO-Ra study, we previously combined peripheral inflammatory indices and clinical factors in a composite score able to stratify the prognosis of these patients since baseline. In the present study, the BIO-Ra score was also a reliable prognostic tool in the current clinical scenario, with a potential added value in the patient’s selection for Radium-223 treatment.

**Abstract:**

The multicentric retrospective BIO-Ra study combined inflammatory indices from peripheral blood and clinical factors in a composite prognostic score for metastatic castration-resistant prostate cancer patients receiving Radium-223 (Ra-223). In the present study, we evaluated (i) the prognostic power of the BIO-Ra score in the framework of the restricted use of Ra-223 promoted by the European Medicines Agency in 2018; (ii) the treatment completion prediction of the BIO-Ra score. Four hundred ninety-four patients from the BIO-Ra cohort were divided into three risk classes according to the BIO-Ra score to predict the treatment completion rate (*p* < 0.001 among all the three groups). Patients receiving Ra-223 after restriction (89/494) were at later stages of the disease compared with the pre-restriction cohort (405/494), as a higher percentage of BIO-Ra high-risk classes (46.1% vs. 34.6%) and lower median Overall survival (12.4 vs. 23.7 months, *p* < 0.001) was observed. Despite this clinically relevant difference, BIO-Ra classes still predicted divergent treatment completion rates in the post-restriction subgroup (72%, 52.2%, and 46.3% of patients belonging to low-, intermediate-, and high-risk classes, respectively). Although the restricted use has increased patients at higher risk with unfavourable outcome after Ra-223 treatment, the BIO-Ra score maintains its prognostic value.

## 1. Introduction

Radium-223 dichloride (Ra-223) is a targeting alpha therapeutic agent that binds areas of high osteoblastic turnover, including bone metastases [1]. The short-range of the emitted alpha particle irradiation allows specific cancer cell targeting, causing double-stranded DNA breaks, and reduces the cytotoxic damage to non-targeted cells [2,3]. In the phase III ALSYMPCA trial [4], metastatic castration-resistant prostate cancer (mCRPC) patients with exclusive bone metastases were randomized between treatment with Ra-223 (experimental arm) or placebo (control arm). The primary endpoint of OS favoured the experimental arm, resulting in 14.9 vs. 11.3 months of median overall survival (mOS) compared with the control arm (HR 0.70; 95% CI 0.58–0.83) with 30% reduction in risk of death. All other secondary efficacy endpoints, such as time to skeletal events, time to increase prostate-specific antigen (PSA), and alkaline phosphatase (ALP), also favoured Ra-223, in the absence of statistically significant differences in terms of haematological adverse events [4]. On these bases, Ra-223 has been rapidly introduced in the clinical setting as a therapeutic option for metastatic castration-resistant prostate cancer (mCRPC) [5,6].

However, in the last decade, clinical practice has reported lower survival benefits than the ALSYMPCA trial results. In fact, different real-life studies described a lower mOS, ranging from 6 to 10 months, compared with the 14.9 months of the ALSYMPCA study [7,8,9,10]. One possible reason for this discrepancy could rely on the suboptimal selection of patients with unfavourable prognostic factors, which might occur in the real-life setting.

Moreover, in 2018, a formal warning promoted by the European Medicines Agency (EMA) limited the Ra-223 prescription to mCRPC patients pre-treated with at least two systemic therapies or ineligible for any systemic treatment and with more than six osteoblastic lesions at bone scan [11]. This moved Ra-223 treatment to the later stages of mCRPC disease, making the patient selection process even more challenging. In this scenario, identifying prognostic factors potentially able to select patients most likely to benefit from Ra-223 since baseline became a crucial clinical issue.

Several studies previously investigated many parameters potentially able to improve patient’s selection since baseline [12,13,14,15,16,17,18]. However, most of them remained inconclusive since none of the identified clinical or biochemical biomarkers has been validated as a unique and reliable selection tool. Based on the preliminary results of a monocentric proof-of-concept study [13], in the multicentric retrospective BIO-Ra study we combined inflammatory indices from peripheral blood and clinical factors in a novel composite prognostic score able to stratify mCRPC patients in three distinctive prognostic groups since baseline, potentially improving the patients’ selection for Ra-223 treatment [19].

In the present analysis, we verified whether the prognostic power of the BIO-Ra score is still preserved in light of the EMA restriction of the use of Ra-223. Moreover, we verified the correlation between the BIO-Ra score and treatment completion, which is a well-known prognostic factor in mCRPC patients treated with Ra-223 [20,21,22,23,24].

## 2. Materials and Methods

The study was performed according to the Declaration of Helsinki, Good Clinical Practice, and local ethical regulations. The study was approved by the local ethical committee of the leading centre (Regional Ethical Committee of Liguria—registration number 535/2020) and then by the local ethical committee of each adhering centre. All patients enrolled in the study signed written informed consent, which included the use of anonymized data for retrospective research purposes.

### 2.1. Study Population and Treatment

The BIO-Ra study is a multicentric retrospective analysis conducted at seven Italian centres collecting clinical data of mCRPC patients receiving at least one cycle of Ra-223 at standard dose in a real-world setting. Detailed inclusion criteria of the BIO-Ra study are reported elsewhere [19]. Briefly, patients must have a diagnosis of mCRPC with symptomatic bone metastases and neither visceral metastases nor lymph nodes >3 cm in short-axis diameter [25]. Ra-223 (50–55 KBq/kg) was intravenously administrated every 4 weeks and was continued until disease progression, death, or patient choice up to six cycles [25].

### 2.2. BIO-Ra Score

The prognostic BIO-Ra score composed by the neutrophil-to-lymphocyte ratio (NLR: < vs. ≥3.1), Eastern Cooperative Oncology Group performance status (ECOG PS: 0–1 vs. 2–3), number of bone metastases (<6 vs. 6–20 vs. ≥20), and alkaline phosphatase (ALP: < vs. ≥220) was assessed before the first cycle of Ra-223 administration. This score identified three distinctive prognostic groups of mCRPC patients: the low-risk group (score 0–2), the intermediate-risk group (score 3–4), and the high-risk group (score 5–10) [19].

### 2.3. Study Endpoints

Among the secondary endpoints of the BIO-Ra study, two endpoints were clinically relevant: (i) the prognostic role of the BIO-Ra score assessed according to the prescription time, before and after the EMA restricted use (July 2018); (ii) the correlation of the BIO-Ra score with treatment completion, defined as the percentage of patients who completed three (T3) and all six cycles (T6). The interplay between these two variables was also assessed to test whether differences among the BIO-Ra risk groups on treatment completion were different before and after the EMA restrictions.

### 2.4. Statistical Analyses

The descriptive analyses were conducted using absolute frequency and percentage for categorical variables and by median and range for quantitative variables. Mann-Whitney test for continuous variables and chi-square test for categorical ones were used to compare characteristics before and after EMA restrictions. The Kaplan-Meier (KM) method was used to estimate the survival curve of OS. To compare OS among the three risk groups detected by the BIO-Ra score, the log-rank test was used. Hazard ratios (HRs) and their 95% confidence intervals (CIs) were estimated by a univariable Cox proportional hazard regression model.

Differences in treatment completion among the risk groups and between the two periods (pre/post EMA restrictions) were assessed using a logistic regression model with follow-up duration considered as an offset to account for different lengths of follow-up. The interaction between the BIO-Ra score and the EMA restriction group was also computed.

All statistical analyses were performed using the software StataCorp. 2019 (Stata Statistical Software: Release 16. StataCorp LLC, College Station, TX, USA).

## 3. Results

### 3.1. Patients’ Characteristics

From September 2013 to July 2020, 519 mCRPC patients receiving Ra-223 were recruited, and in 494 patients (95%) the BIO-Ra score was calculable. Patients’ and treatment characteristics are summarized in Table 1. The median age was 74 years (50–90 years), and patients older than ≥75 years were 48% of the entire cohort. At baseline, most patients had an ECOG PS of 0–1 (76.7%), no lymph nodal metastases (65.6%), and a number of bone metastases between 6 and 20 (57.9%). Among all patients, 47.6%, 38.5%, and 13.9% received Ra-223 as 1st–2nd, 3rd–4th, and further-line, respectively. Most of them had also previously received chemotherapy (61.5%).

Among the 494 patients, 405 patients (82%) received Ra-223 treatment before the EMA restricted use, while 89 patients (15%) after this time (Table 1). At the time of data cut-off (February 2021), with a median follow-up of 10.7 months, 88.7% (*n* = 438) of patients completed T3, and 62.2% (*n* = 307) of patients completed T6.

### 3.2. EMA Restricted Use

#### 3.2.1. Population and BIO-Ra Score According to EMA Restrictions

**Figure 1:**
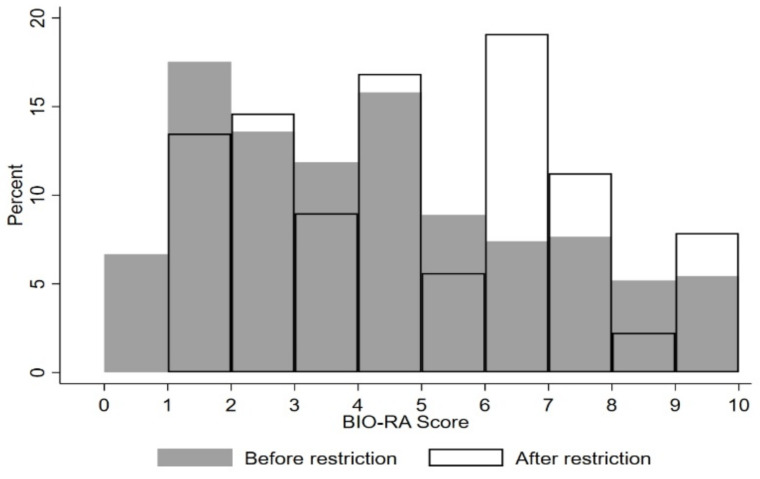
The BIO-Ra scores distribution before and after EMA restrictions. Each column describes the frequency of the corresponding BIO-Ra score in the study cohort. Full and empty columns correspond to patients enrolled before and after EMA restrictions, respectively.

Patients receiving Ra-223 after the EMA restriction were at later stages of the disease compared with the pre-restriction cohort, as suggested by the higher frequency of lymphadenopathies, higher number of bone metastases, and previously administered chemotherapy, as well as by the higher PSA and lower haemoglobin levels (Table 1). The BIO-Ra score distribution showed a slight but statistically significant difference (*p* = 0.013) between the two periods (before/after EMA restrictions) (Figure 1). After the EMA restriction, a higher percentage of patients belonged to the high-risk group (Score 5–10) (46.1% vs. 34.6%) and a lower percentage to the low-risk group (Score: 0–2) (28.1% vs. 37.8%).

#### 3.2.2. Correlation between OS and EMA Restrictions, According to the BIO-Ra Score

**Figure 2:**
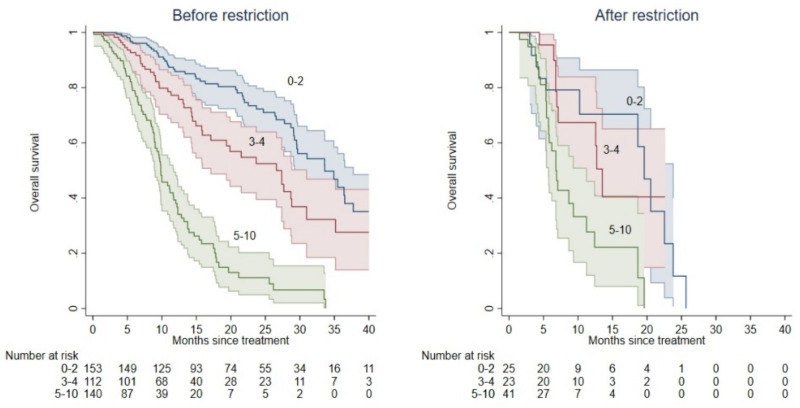
Kaplan-Meier OS curves according to the BIO-Ra risk classes before and after the EMA restriction of the use of Ra-223. OS prediction according to the BIO-Ra score, categorizing patients into three prognostic groups before (**left Panel**) and after (**right Panel**) the EMA restriction of the use of Ra-223. BIO-Ra scores 0–2, 3–4, and 5–10 corresponding to low-, intermediate-, and high-risk classes are represented as blue, red, and green colours, respectively.

Patients treated with Ra-223 after the EMA restriction showed lower mOS compared with those treated before the restricted use (12.4 vs. 23.7 months, *p* < 0.001, Figure 2).

Among the 405 patients treated before the EMA restriction, mOS was 33.6 (CI: 29.5–37.7), 26.6 (CI: 17.1–31.0), and 9.8 (CI: 8.9–11.7) for patients belonging to the low, intermediate, and high-risk group according to the BIO-Ra score. Patients in the intermediate-risk (HR = 1.73; 95% CI: 1.17–2.55; *p* = 0.006) and high-risk groups (HR = 6.38; 95% CI: 4.41–9.22; *p* < 0.001) had a significantly lower mOS as compared with those in the low-risk group. Moreover, patients in the high-risk group had a worse mOS as compared with the intermediate-risk group (HR = 3.68; 95% CI: 2.52–5.37; *p* < 0.001).

By contrast, mOS resulted in 19.6 (CI: 10.2–23.8), 13.5 (CI: 6.9–21.0), and 6.8 (CI: 5.7–11.2) in patients belonging to the low, intermediate, and high-risk group according to the BIO-Ra score in the remaining 89 patients receiving Ra-223 after the EMA restriction. In this cohort of patients, the mOS was not significantly different between low- and intermediate-risk groups (HR = 1.08; 95% CI: 0.43–2.69; *p* = 0.87), while it was significantly lower in the high-risk group (vs. 1: HR = 3.10; 95% CI: 1.42–6.77; *p* = 0.005; vs. 2: HR = 2.87; 95% CI: 1.32–6.23; *p* = 0.008).

### 3.3. Treatment Completion

#### 3.3.1. Correlation between OS and Treatment Completion

**Figure 3:**
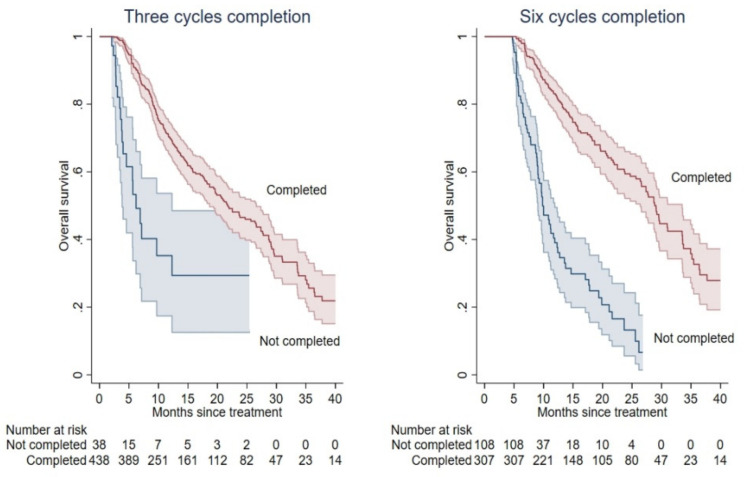
Kaplan-Meier OS curves according to treatment completion. OS prediction according to treatment completion at T3 (**left panel**) and T6 (**right panel**). Completed and incomplete cycles are represented as red and blue colours, respectively.

Among all patients, only 18 patients (4%) had not enough follow-up to reach the timepoint for the third cycle administration. Among the remaining 476 patients (96%) with at least two months of follow-up, patients who completed T3 (N = 438, 92%) were associated with longer mOS compared with those who did not (21.8 vs. 6.2 months; *p* < 0.001; Figure 3). A total of 415 patients (84%), instead, reached the timepoint of 5 months for the sixth cycle administration. Among these, the 307 patients (74%) who completed T6 were associated with longer mOS compared with those who did not (29.1 vs. 9.8; *p* < 0.001; Figure 3).

#### 3.3.2. Treatment Completion According to the BIO-Ra Score

The BIO-Ra low- and intermediate-risk groups were associated with a statistically higher percentage of treatment completion T3 compared with the high-risk group (98.8% vs. 94.1% vs. 74.6%, respectively; *p* < 0.001). According to the completion T6, the more favourable was the prognostic group, the higher was the percentage of treatment completion, with significant differences among all the three groups (84.3% of low-risk group vs. 68.9% of intermediate-risk group and 35.4% of high-risk group; *p* < 0.001).

#### 3.3.3. Correlation between Treatment Completion and EMA Restrictions

Patients treated after the EMA restricted use were associated with lower rate of treatment completion, especially at T6, without statistically significant differences (T3: 86.5% vs. 89.1% *p* = 0.86; T6: 55.1% vs. 63.7% *p* = 0.12) compared with patients treated before the EMA restricted use.

#### 3.3.4. Correlation of BIO-Ra Score, Treatment Completion, and EMA Restrictions

After the EMA amendment, the BIO-Ra risk classes still predicted divergent treatment completion rates. Regarding the completion T3, results according to the BIO-Ra score were similar also after splitting according to the EMA restricted use (*p* for interaction BIO-Ra score—EMA restriction group = 0.53). For the completion T6, instead, statistically significant differences between the two periods according to the BIO-Ra score were observed (*p* for interaction BIO-Ra score—EMA restriction group = 0.013). In fact, before the EMA restriction, the completion T6 was observed in 86.3%, 72.3%, and 32.1% of patients belonging to the low-, intermediate-, and high-risk groups, respectively. By contrast, intermediate- and high-risk groups showed very similar (52.2% and 46.3%) treatment completion rates at T6, instead of the low-risk group (72%).

## 4. Discussion

In 2018, based on the review of the ERA-223 trial [26], the EMA’s Pharmacovigilance Risk Assessment Committee issued a formal warning against using Ra-223 in combination with abiraterone acetate plus prednisone/prednisolone in patients with mCRPC [11]. However, EMA retraction concerned not only the combination of Ra-223 with androgen receptor-targeted agents but also its prescription as monotherapy [11,27]. Indeed, the presence of progressive disease after at least two previous treatments for mCRPC was set as a new precondition for the prescription of Ra-223. In the clinical setting, the use of Ra-223 was inevitably limited to the later stages of the disease, making more challenging the patient’s selection for this treatment, leading to the urgent need to identify reliable biomarkers whose prognostic power could be still preserved in light of the EMA restriction.

The BIO-Ra multicentric retrospective study developed a widely applicable integrated score combining performance status, tumour burden, and systemic inflammation, which is able to identify subgroups of patients with distinctive survival outcomes following Ra-223 administration [19]. The main aim of the present study was to assess the capability of the BIO-Ra score to predict treatment completion rates, especially in the subgroup of patients treated after EMA restriction.

In the present analysis, we observed a higher frequency of patients with unfavourable prognostic characteristics such as pre-treatment with chemotherapy, lymphadenopathies, high bone metastatic tumour burden (depicted by the number of bone lesions at the bone scan and PSA levels), and low haemoglobin levels in patients treated with Ra-223 after the EMA amendment. Notably, the more advanced disease was paralleled by lower mOS, which was roughly halved compared with patients treated before the restriction, coherent with the previous literature [28]. Consistently, we observed that in the post-EMA restriction period a higher percentage of patients belonged to the higher BIO-Ra risk class. Of note, this subgroup of patients showed lower OS compared with the remaining subgroups, suggesting that the prognostic value of the BIO-Ra score is preserved in the present clinical setting. This finding is coherent with the observed prediction of treatment completion by the BIO-Ra score in the same time interval. Indeed, several previous studies showed a substantial survival benefit in patients reaching the completion of Ra-223 cycles [20,21,22,23,24].

The most likely cause of the poor prognosis in patients receiving incomplete Ra-223 treatment is the advanced stage of their disease. However, it can be hypothesized that the Ra-223 effective dose is only achieved by giving all the six cycles and that anything less is undertreatment, as was suggested in the latest joint procedural guidelines by the Committee on Practice Parameters of the American College of Radiology (ACR) in collaboration with the American College of Nuclear Medicine (ACNM), the American Society for Radiation Oncology (ASTRO), and the Society of Nuclear Medicine and Molecular Imaging (SNMMI) [29].

Of note, while mean treatment completion rates were not significantly different between patients treated before and after the EMA restriction, differences between the two periods according to the BIO-Ra risk stratification were observed. In particular, while treatment completion rates were largely different before the EMA amendment between the three BIO-Ra risk classes, in the post-restriction era, patients belonging to intermediate- and high-risk groups showed remarkably similar treatment completions. On the one side, this finding might be related to the lower statistical power of the post-restriction study cohort. On the other side, whether confirmed in a larger and independent patient cohort, it might suggest that caution is also needed when treating patients at intermediate-risk, underlining the need to revise the current Ra-223 treatment selection criteria to maximize the benefit of this therapy in mCRPC patients.

The present study has some limitations. First, the retrospective nature of the analysis may inevitably introduce a selection bias related to the heterogeneous diagnostic and therapeutic approach of each adhering centre. Therefore, although only high-volume tertiary-level institutions participated in the BIO-Ra study and the current international guidelines were followed [25], independent external validation is mandatory to confirm the robustness of the obtained results. Second, as already stated above, the post-restriction cohort had a smaller size than the pre-restriction one, with a relatively shorter follow-up. Therefore, dedicated analyses with longer follow-up are needed to confirm our results. Third, although the present data suggest the capability of the BIO-Ra score to identify patients who would most benefit from Ra-223 since baseline, the validation of the BIO-Ra risk stratification as a supporting tool for the patient selection process needs external validation in a prospective trial. Finally, the present study lacks the use of molecular biomarker data. In recent years, several bio-molecular alterations have been observed in PC, showing a potential predictive power for treatment planning [6]. In a precision medicine perspective, the addition of molecular biomarkers to the BIO-Ra score might further improve its capability for selecting the best drug for the right patient. A similar consideration applies to next-generation imaging, particularly with positron emission tomography (PET), whose functional nature may at the same time provide an accurate staging of the disease extent [30] and display tumour heterogeneity in vivo [13,17,18,31,32,33,34,35], potentially improving the patient selection process. Unfortunately, given the study’s retrospective nature, we built the BIO-Ra score exclusively using widely available clinical and lab data. However, this also represents the strength of our approach, as it makes the BIO-Ra score easily and widely applicable for clinical practice at no additional costs.

## 5. Conclusions

The present study showed that the 2018 EMA restriction of the use of Ra-223 has led to treating mCRPC patients at a later stage of the disease, with a measurable impact on survival outcomes. In this context, the BIO-Ra score was demonstrated to be a reliable prognostic tool also in the current clinical scenario, with a potential added value in patient selection for Ra-223 treatment.

## Figures and Tables

**Table 1:** Patients’ characteristics.

Characteristics	All Patients	Before EMA Restricted Use	After EMA Restricted Use	*p* Value
	*n* = 494	*n* = 405	*n* = 89	
	*n* (%)			
Age, years Median (range)	74 (50–90)	74 (50–90)	75 (52–89)	0.465
<75	257 (52.0)	212 (52.4)	45 (50.6)	0.760
≥75	237 (48.0)	193 (47.7)	44 (49.4)	
ECOG PS				
Median (range)	1 (0–3)	1 (0–3)	1 (0–3)	0.409
0–1	379 (76.7)	313 (77.3)	66 (74.2)	0.527
2–3	115 (23.3)	92 (22.7)	23 (25.8)	
Gleason score				
Median (range)	8 (5–10)	8 (5–10)	8 (6–10)	0.892
≤7	166 (33.6)	135 (33.3)	31 (34.8)	0.959
≥8	249 (50.4)	203 (50.1)	46 (51.7)	
Missing	79 (16.0)	67 (16.5)	12 (13.5)	
Lymphadenopathies				
Yes	156 (31.6)	119 (29.4)	37 (41.6)	0.063
No	297 (60.1)	248 (61.2)	49 (55.1)	
Missing	41 (8.3)	38 (9.4)	3 (3.4)	
Number of bone metastases				
<6	60 (12.2)	56 (13.8)	4 (4.5)	<0.001
6–20	286 (57.9)	243 (60.0)	43 (48.3)	
>20	148 (30.0)	106 (26.2)	42 (47.2)	
Ra-223 treatment line				
Median (range)	3 (1–9)	2 (1–9)	3 (1–9)	0.001
First and second line	235 (47.6)	208 (51.4)	27 (30.3)	<0.001
≥Third line	259 (52.4)	197 (48.6)	62 (69.7)	
Prior chemotherapy				
Yes	304 (61.5)	239 (59.0)	65 (73.0)	0.014
No	190 (38.5)	166 (41.0)	24 (27.0)	
Bisphosphonates/Denosumab use				
Yes	224 (45.3)	178 (44.0)	46 (51.7)	0.107
No	267 (54.1)	227 (56.1)	40(44.9)	
Missing	3 (0.6)	0(0.0)	3 (3.4)	
Baseline ALP, U/L				
Median (range)	145 (0–2474)	142 (0–1798)	149 (36–2474)	0.744
<220	330 (66.8)	273 (67.4)	57 (64.0)	0.542
≥220	164 (33.2)	132 (32.6)	32 (36.0)	
Baseline median LDH, U/L (range)	295 (129–2146)	309.5 (129–2146)	266 (152–988)	0.038
Baseline median PSA, ng/mL (range)	58.6 (0.03–6089)	53.3 (0.03–3000)	85.3 (0.39–6089)	0.024
Baseline median Hb, g/dL (range)	12.2 (7.8–15.9)	12.3 (8.1–15.9)	11.5 (7.8–15)	0.007

*n*, number; ECOG, Eastern Cooperative Oncology Group; PS, performance status; EMA, European Medicines Agency; ALP, alkaline phosphatase; LDH, lactate dehydrogenase; PSA, prostate-specific antigen; Hb, haemoglobin.

## Data Availability

The data presented in this study are available on request from the corresponding author.

## References

[1] De Vincentis G., Gerritsen W., Gschwend J.E., Hacker M., Lewington V., O’Sullivan J.M., Oya M., Pacilio M., Parker C., Shore N. (2019). Advances in targeted alpha therapy for prostate cancer. Ann. Oncol..

[2] Kassis A.I., Adelstein S.J. (2005). Radiobiologic principles in radionuclide therapy. J. Nucl. Med..

[3] Harrison M.R., Wong T.Z., Armstrong A.J., George D.J. (2013). Radium-223 chloride: A potential new treatment for castration-resistant prostate cancer patients with metastatic bone disease. Cancer Manag. Res..

[4] Parker C., Nilsson S., Heinrich D., Helle S.I., O’Sullivan J.M., Fosså S.D., Chodacki A., Wiechno P., Logue J., Seke M. (2013). Alpha emitter radium-223 and survival in metastatic prostate cancer. N. Engl. J. Med..

[5] Kluetz P.G., Pierce W., Maher V.E., Zhang H., Tang S., Song P., Liu Q., Haber M.T., Leutzinger E.E., Al-Hakim A. (2014). Radium Ra 223 dichloride injection: U.S. Food and Drug Administration drug approval summary. Clin. Cancer Res..

[6] Cattrini C., España R., Mennitto A., Bersanelli M., Castro E., Olmos D., Lorente D., Gennari A. (2021). Optimal Sequencing and Predictive Biomarkers in Patients with Advanced Prostate Cancer. Cancers.

[7] Parikh S., Murray L., Kenning L., Bottomley D., Din O., Dixit S., Ferguson C., Handforth C., Joseph L., Mokhtar D. (2018). Real-world Outcomes and Factors Predicting Survival and Completion of Radium 223 in Metastatic Castrate-resistant Prostate Cancer. Clin. Oncol..

[8] Wong W.W., Anderson E.M., Mohammadi H., Daniels T.B., Schild S.E., Keole S.R., Choo C.R., Tzou K.S., Bryce A.H., Ho T.H. (2017). Factors Associated with Survival Following Radium-223 Treatment for Metastatic Castration-resistant Prostate Cancer. Clin. Genitourin. Cancer.

[9] Kuppen M.C., Westgeest H.M., van der Doelen M.J., van den Eertwegh A.J., Coenen J.L., Aben K.K., van den Bergh A.C., Bergman A.M., den Bosch J.V., Celik F. (2020). Real-world outcomes of radium-223 dichloride for metastatic castration resistant prostate cancer. Future Oncol..

[10] Frantellizzi V., Farcomeni A., Follacchio G.A., Pacilio M., Pellegrini R., Pani R., De Vincentis G. (2018). A 3-variable prognostic score (3-PS) for overall survival prediction in metastatic castration-resistant prostate cancer treated with 223Radium-dichloride. Ann. Nucl. Med..

[11] EMA EMA Restricts Use of Prostate Cancer Medicine XOFIGO. https://www.ema.europa.eu/en/news/ema-restricts-use-prostate-cancer-medicine-xofigo.

[12] Laudicella R., Albano D., Alongi P., Argiroffi G., Bauckneht M., Baldari S., Bertagna F., Boero M., Vincentis G., Sole A.D. (2019). 18F-Facbc in Prostate Cancer: A Systematic Review and Meta-Analysis. Cancers.

[13] Bauckneht M., Rebuzzi S.E., Signori A., Donegani M.I., Murianni V., Miceli A., Borea R., Raffa S., Damassi A., Ponzano M. (2020). The Prognostic Role of Baseline Metabolic Tumor Burden and Systemic Inflammation Biomarkers in Metastatic Castration-Resistant Prostate Cancer Patients Treated with Radium-223: A Proof of Concept Study. Cancers.

[14] Al-Ezzi E.M., Alqaisi H.A., Iafolla M., Wang L., Sridhar S.S., Sacher A.G., Fallah-Rad N., Jiang D.M., Watson G.A., Catton C.N. (2021). Clinicopathologic factors that influence prognosis and survival outcomes in men with metastatic castration-resistant prostate cancer treated with Radium-223. Cancer Med..

[15] Van der Zande K., Oyen W.J.G., Zwart W., Bergman A.M. (2021). Radium-223 Treatment of Patients with Metastatic Castration Resistant Prostate Cancer: Biomarkers for Stratification and Response Evaluation. Cancers.

[16] Frantellizzi V., Monari F., Mascia M., Costa R., Rubini G., Spanu A., Di Rocco A., Lodi Rizzini E., Cindolo L., Licari M. (2020). Validation of the 3-variable prognostic score (3-PS) in mCRPC patients treated with 223Radium-dichloride: A national multicenter study. Ann. Nucl. Med..

[17] Bauckneht M., Capitanio S., Donegani M.I., Zanardi E., Miceli A., Murialdo R., Raffa S., Tomasello L., Vitti M., Cavo A. (2019). Role of Baseline and Post-Therapy 18F-FDG PET in the Prognostic Stratification of Metastatic Castration-Resistant Prostate Cancer (mCRPC) Patients Treated with Radium-223. Cancers.

[18] Bauckneht M., Lai R., D’Amico F., Miceli A., Donegani M.I., Campi C., Schenone D., Raffa S., Chiola S., Lanfranchi F. (2022). Opportunistic skeletal muscle metrics as prognostic tools in metastatic castration-resistant prostate cancer patients candidates to receive Radium-223. Ann. Nucl. Med..

[19] Bauckneht M., Rebuzzi S.E., Signori A., Frantellizzi V., Murianni V., Lodi Rizzini E., Mascia M., Lavelli V., Donegani M.I., Ponzano M. (2022). The prognostic power of inflammatory indices and clinical factors in metastatic castration-resistant prostate cancer patients treated with radium-223 (BIO-Ra study). Eur. J. Nucl. Med. Mol. Imaging.

[20] Buscombe J., Gillett D., Bird N., Powell A., Heard S., Aloj L. (2020). Quantifying the survival benefit of completing all the six cycles of radium-223 therapy in patients with castrate-resistant prostate cancer with predominant bone metastases. World J. Nucl. Med..

[21] Dadhania S., Alonzi R., Douglas S., Gogbashian A., Hughes R., Dalili D., Vasdev N., Adshead J., Lane T., Westbury C. (2018). Single-centre experience of use of radium 223 with clinical outcomes based on number of cycles and bone marrow toxicity. Anticancer Res..

[22] Uemura H., Uemura H., Nagamori S., Wakumoto Y., Kimura G., Kikukawa H., Yokomizo A., Mizokami A., Kosaka T., Masumori N. (2019). Three-year follow-up of a phase II study of radium-223 dichloride in Japanese patients with symptomatic castration-resistant prostate cancer and bone metastases. Int. J. Clin. Oncol..

[23] Sasaki D., Hatakeyama S., Kawaguchi H., Hatayama Y., Ishibashi Y., Kusaka A., Noro D., Tanaka T., Ito H., Okuyama Y. (2022). Effects of six-cycle completion and earlier use of radium-223 therapy on prognosis for metastatic castration-resistant prostate cancer: A real-world multicenter retrospective study. Urol. Oncol. Semin. Orig. Investig..

[24] Cheng S., Arciero V., Goldberg H., Tajzler C., Manganaro A., Kozlowski N., Rowbottom L., McDonald R., Chow R., Vasisht G. (2019). Population-based analysis of the use of radium-223 for bone-metastatic castration-resistant prostate cancer in Ontario, and of factors associated with treatment completion and outcome. Cancer Manag. Res..

[25] Poeppel T.D., Handkiewicz-Junak D., Andreeff M., Becherer A., Bockisch A., Fricke E., Geworski L., Heinzel A., Krause B.J., Krause T. (2018). EANM guideline for radionuclide therapy with radium-223 of metastatic castration-resistant prostate cancer. Eur. J. Nucl. Med. Mol. Imaging.

[26] Smith M., Parker C., Saad F., Miller K., Tombal B., Ng Q.S., Boegemann M., Matveev V., Piulats J.M., Zucca L.E. (2019). Addition of radium-223 to abiraterone acetate and prednisone or prednisolone in patients with castration-resistant prostate cancer and bone metastases (ERA 223): A randomised, double-blind, placebo-controlled, phase 3 trial. Lancet Oncol..

[27] Van den Wyngaert T., Tombal B. (2019). The changing role of radium-223 in metastatic castrate-resistant prostate cancer: Has the EMA missed the mark with revising the label?. Q. J. Nucl. Med. Mol. Imaging.

[28] Jarvis P., Ho A., Sundram F. (2021). Radium-223 therapy for metastatic castration-resistant prostate cancer: Survival benefit when used earlier in the treatment pathway. Nucl. Med. Commun..

[29] Hurwitz M., Buscombe J.R., Jacene H.A., Klitzke A.K., Lamonica D., Lu Y., Pryma D.A., Rohren E.M., Speer T.W., Subramaniam R.M. (2020). ACR-ACNM-ASTRO-SNMMI practice parameter for the performance of therapy with radium-223. Am. J. Clin. Oncol..

[30] Borea R., Favero D., Miceli A., Donegani M.I., Raffa S., Gandini A., Cremante M., Marini C., Sambuceti G., Zanardi E. (2022). Beyond the Prognostic Value of 2-[^18^F]FDG PET/CT in Prostate Cancer: A Case Series and Literature Review Focusing on the Diagnostic Value and Impact on Patient Management. Diagnostics.

[31] Bauckneht M., Bertagna F., Donegani M.I., Durmo R., Miceli A., De Biasi V., Laudicella R., Fornarini G., Berruti A., Baldari S. (2021). The prognostic power of 18F-FDG PET/CT extends to estimating systemic treatment response duration in metastatic castration-resistant prostate cancer (mCRPC) patients. Prostate Cancer Prostatic Dis..

[32] Filippi L., Spinelli G.P., Chiaravalloti A., Schillaci O., Equitani F., Bagni O. (2020). Prognostic Value of 18F-Choline PET/CT in Patients with Metastatic Castration-Resistant Prostate Cancer Treated with Radium-223. Biomedicines.

[33] García Vicente A.M., González García B., Amo-Salas M., García Carbonero I., Cassinello Espinosa J., Gómez-Aldaraví Gutierrez J.L., Suarez Hinojosa L., Soriano Castrejón Á. (2019). Baseline 18F-Fluorocholine PET/CT and bone scan in the outcome prediction of patients treated with radium 223 dichloride. Clin. Transl. Oncol..

[34] Vija Racaru L., Sinigaglia M., Kanoun S., Ben Bouallègue F., Tal I., Brillouet S., Bauriaud-Mallet M., Zerdoud S., Dierickx L., Vallot D. (2018). Fluorine-18-fluorocholine PET/CT parameters predictive for hematological toxicity to radium-223 therapy in castrate-resistant prostate cancer patients with bone metastases: A pilot study. Nucl. Med. Commun..

[35] Letellier A., Johnson A.C., Kit N.H., Savigny J.F., Batalla A., Parienti J.J., Aide N. (2018). Uptake of Radium-223 Dichloride and Early [^18^F]NaF PET Response Are Driven by Baseline [^18^F]NaF Parameters: A Pilot Study in Castration-Resistant Prostate Cancer Patients. Mol. Imaging Biol..

